# Tau Disaggregation by a CNS-Permeable Small Molecule Reduces Fibril and Oligomer Burden and Preserves Proteostasis and Behavior

**DOI:** 10.64898/2026.06.09.731185

**Published:** 2026-06-12

**Authors:** N Mostowfi, R Foreman, J Wang, R Khoury, A Albanese, QL Ma, W Cohn, G Petzinger, M Jakowec, SK Ahmed, PM Seidler

## Abstract

Pathological tau aggregates drive neuronal dysfunction in Alzheimer’s disease (AD) and related tauopathies, yet no approved therapy eliminates existing tau neurofibrillary tangles. Here, we report the development of a coumarin-based small-molecule series that disaggregates tau fibrils and oligomers through a stacking-driven co-assembly mechanism. Structure-activity relationships identified PT-13 as a lead compound that inhibits tau seeding by AD brain-derived matter and reduces aggregate burden measured across both fibrillar and oligomeric tau species. Mechanistic studies demonstrate that disaggregation does not generate soluble oligomeric intermediates, addressing a central question in the field. PT-13 is brain-penetrant and well tolerated in vivo. In a tauopathy mouse model, PT-13 treatment reduces tau pathology while preserving behavioral function, proteasome capacity, and synaptic integrity. These findings establish small-molecule tau disaggregation as a viable therapeutic strategy and provide a molecular framework for the design of aggregate-directed therapeutics in neurodegeneration.

## Full Text Availability

The license terms selected by the author(s) for this preprint version do not permit archiving in PMC. The full text is available from the preprint server.

