# Supplementary material for: Tau Disaggregation by a CNS-Permeable Small Molecule Reduces Fibril and Oligomer Burden and Preserves Proteostasis and Behavior
Source: bioRxiv. 2026 Sep 1:2026.06.09.731185. Originally published 2026 Jun 12. Preprint. [Version 2] doi: 10.64898/2026.06.09.731185 (PMC13277982; doi:10.64898/2026.06.09.731185)
Supplement: Supplementary file 1 [file media-1.pdf]

Tau Disaggregation by a CNS-Permeable Small Molecule Reduces Fibril and Oligomer Burden and Preserves Proteostasis and Behavior

Mostowfi N, Foreman R, Wang J, Khoury R, Albanese A, Ma QL, Cohn W, Petzinger G, Jakowec M, Ahmed SK, Seidler PM

Supplementary Figures

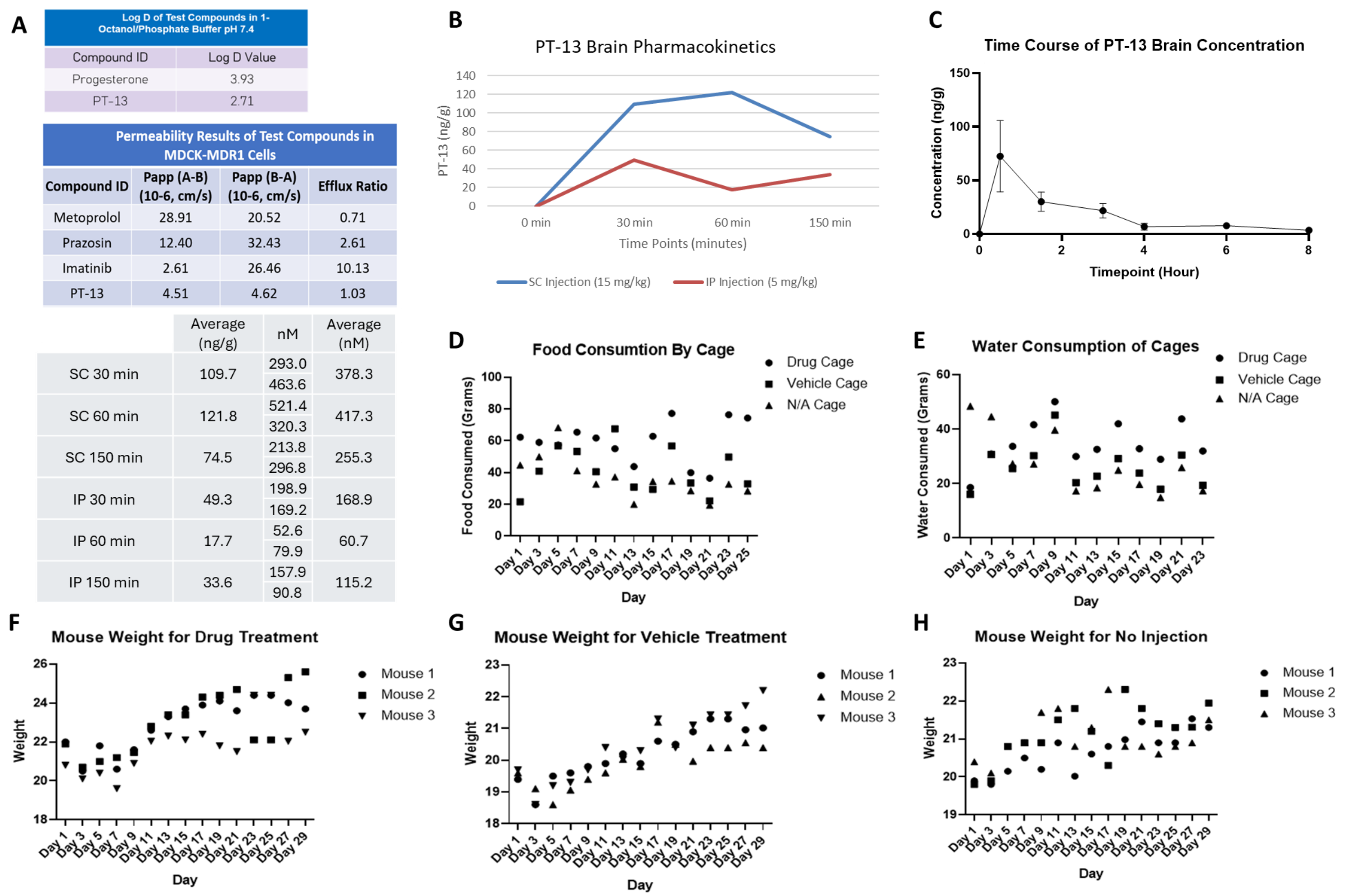

Log D of Test Compounds in 1-Octanol/Phosphate Buffer pH 7.4

Compound ID	Log D Value
Progesterone	3.93
PT-13	2.71

Permeability Results of Test Compounds in MDCK-MDR1 Cells

Compound ID	Papp (A-B) (10-6, cm/s)	Papp (B-A) (10-6, cm/s)	Efflux Ratio
Metoprolol	28.91	20.52	0.71
Prazosin	12.40	32.43	2.61
Imatinib	2.61	26.46	10.13
PT-13	4.51	4.62	1.03

	Average (ng/g)	nM	Average (nM)
SC 30 min	109.7	293.0<br>463.6	378.3
SC 60 min	121.8	521.4<br>320.3	417.3
SC 150 min	74.5	213.8<br>296.8	255.3
IP 30 min	49.3	198.9<br>169.2	168.9
IP 60 min	17.7	52.6<br>79.9	60.7
IP 150 min	33.6	157.9<br>90.8	115.2

Supplementary Figure 1. Pilot data demonstrating PT-13 BBB permeability and dosage tolerability. (A) In vitro ADMET data. PT-13 demonstrates an efflux ratio of 1.0 in MDCK-MDR1 cells similar to positive control compounds, Metoprolol and Prazosin, indicating effective membrane crossing and stable retention in cells. Log D determination in 1-Octanol/Phosphate Buffer, pH 7.4, indicates excellent cell permeability compared to control molecule, Progesterone. (B) Initial BBB permeability pilot. Mice injected with highest possible dose through subcutaneous injection (SC) or intraperitoneal injection (IP). Injected dose was 5 mg/kg PT-13 by IP and 15 mg/kg SC. PT-13 from brain tissue was quantified by MRM mass spectrometry. C57BL/6J mice n = 2 per timepoint for each injection site. (C) Extended BBB permeability measures for preferred injection route. C57BL/6J mice were injected with a single subcutaneous dose of 15 mg/kg PT-13. Brain concentrations of PT-13 measured at the indicated time points post-injection are shown (n = 3 female mice per time point). (D-H) Dosage tolerance was assessed in PT-13 and control animals by monitoring food and water intake, D and E, in C57BL/6J administered at 15 mg/kg PT-13 was well tolerated over 29 days. No observable signs of toxicity were seen. F-H, mice continued normal weight gain throughout the pilot study for PT-13 and vehicle treatment.

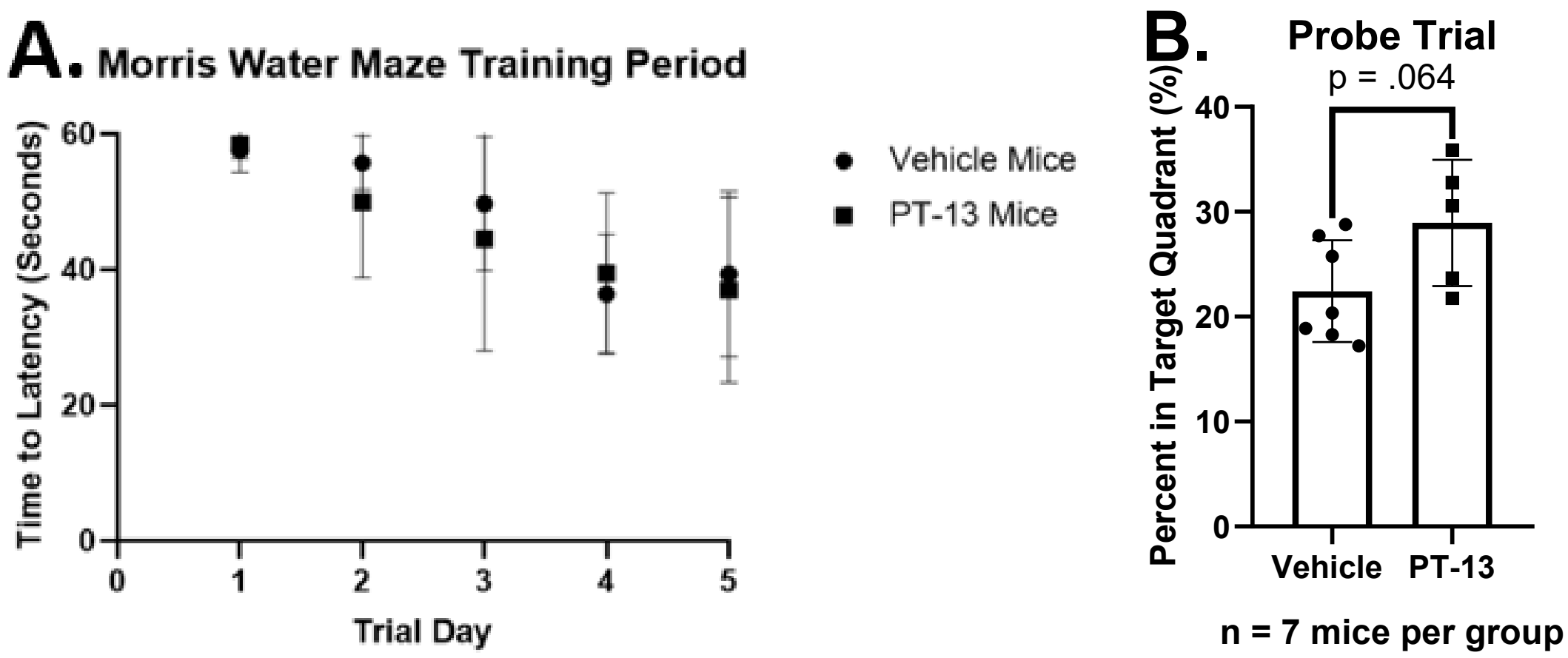

Supplemental Figure 2: Drug treatment induces no statistically significant difference in the time of latency for locating the platform in the Morris Water Maze.

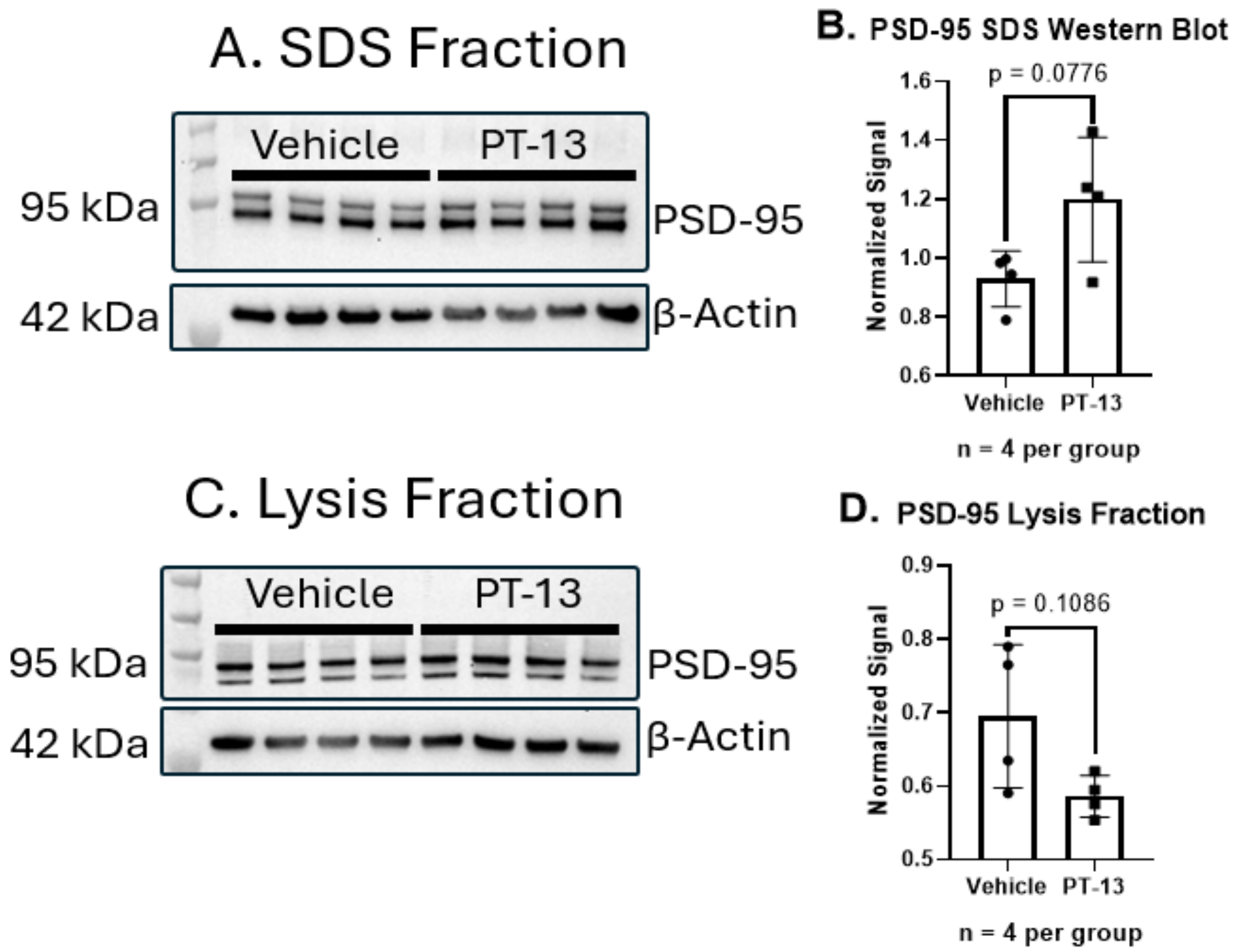

Supplemental Figure 3: PT-13 treatment shows a trending shift in PSD-95 concentration between the SDS and Lysis fractions extracted from the cortex tissue of rTg4510 mice. PSD-95 signal was normalized to beta-actin.

Supplementary Tables

Table 1: Biological Activity Data for Compounds M1-M40

Compound ID	Structure	Molecular Weight	Biological Data
**M1 'CNS-16'**	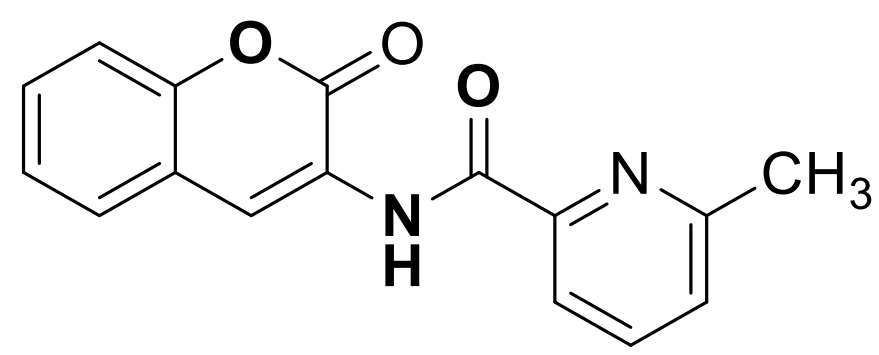	280.28	20% Inhibition against AD Seeding on average
**M2 'PT-31'**	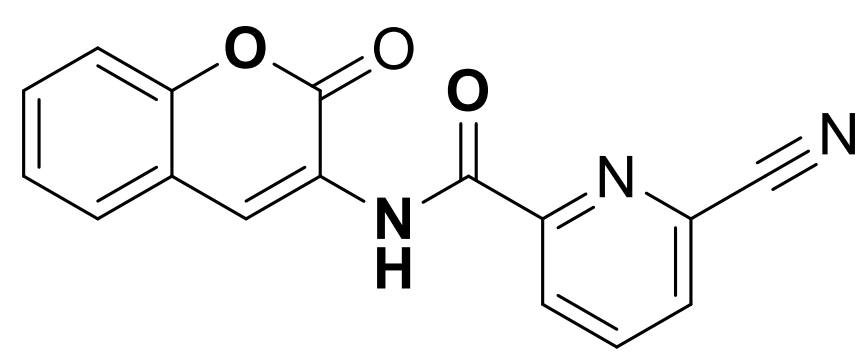	291.26	60% Inhibition against AD Seeding on average
**M3**	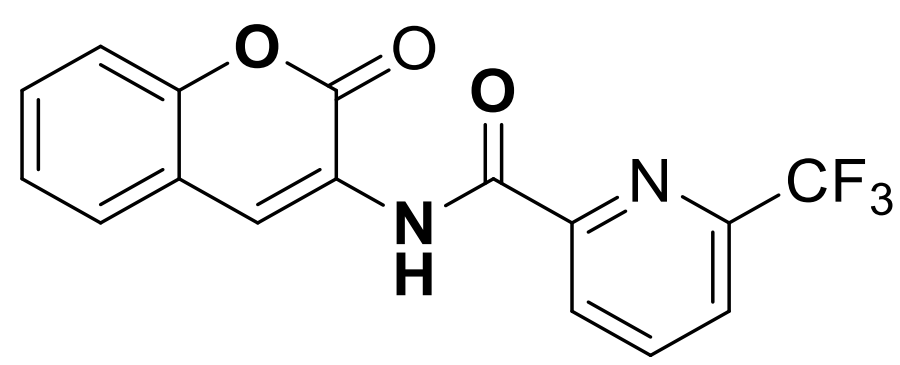	334.25	30% Increase in AD Seeding on average
**M4 'PT-13'**	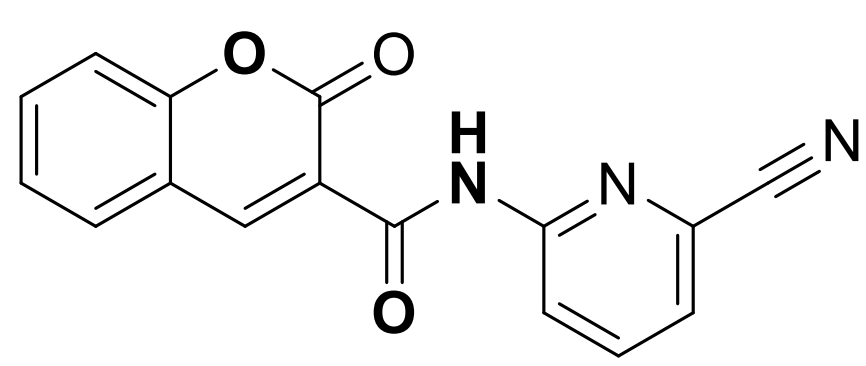	291.26	85% Inhibition against AD Seeding on average
**M5 'PT-92'**	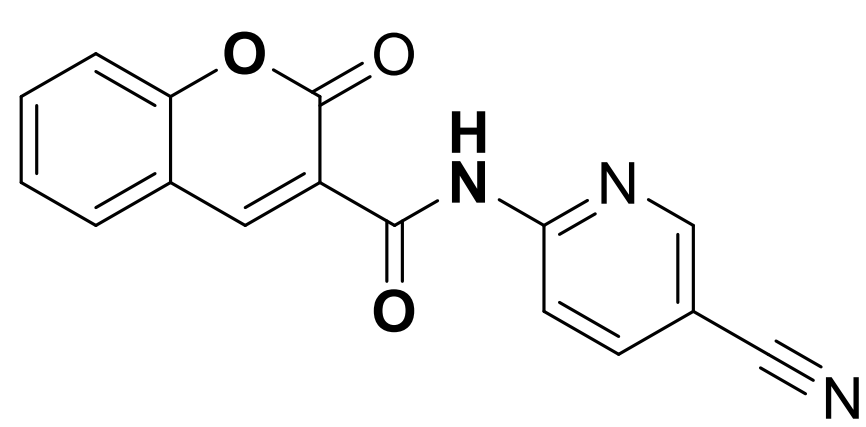	291.26	50% Inhibition against AD Seeding on average
**M6 'PT-97'**	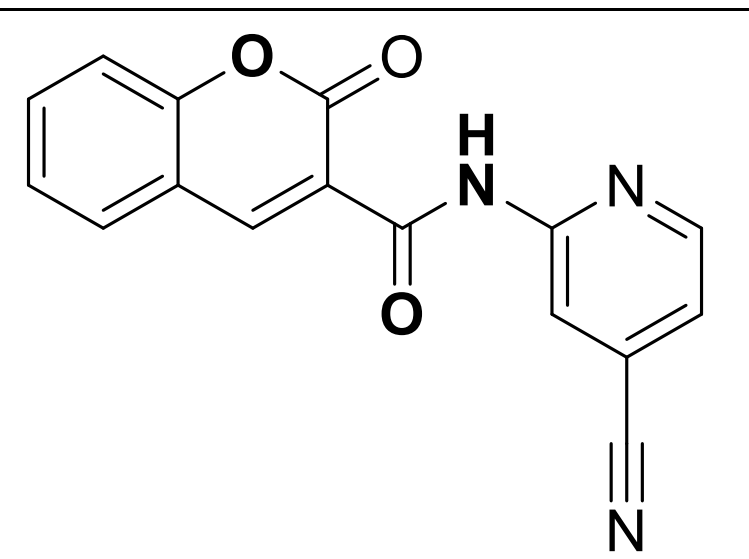	291.26	No notable effect on AD seeding

Compound ID	Structure	Molecular Weight	Biological Data
**M7**	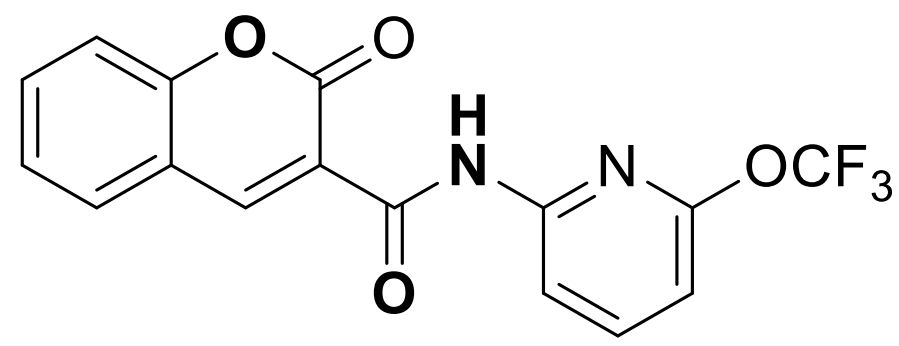	350.25	25% Inhibition against AD Seeding on average
**M8**	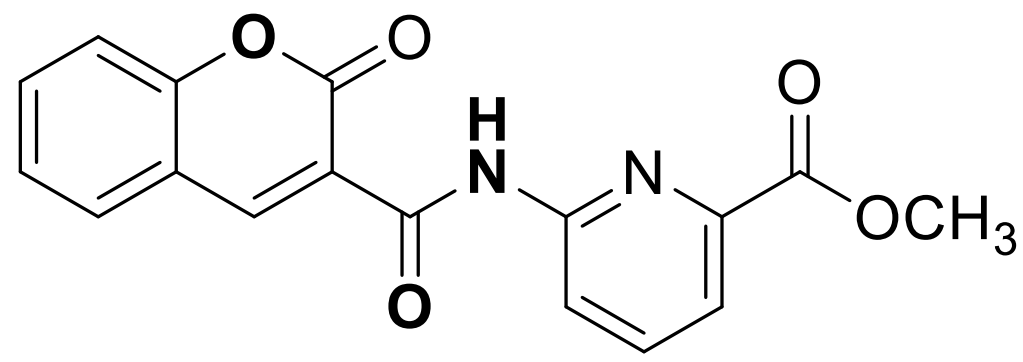	324.29	No notable effect on AD seeding
**M9**	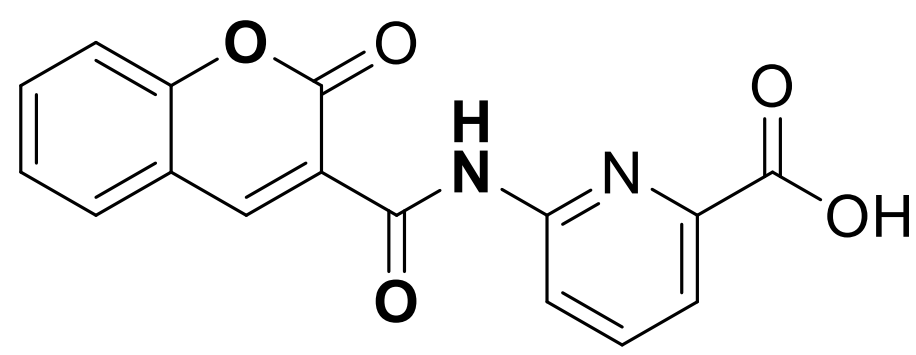	310.26	30% Inhibition against AD Seeding on average
**M10**	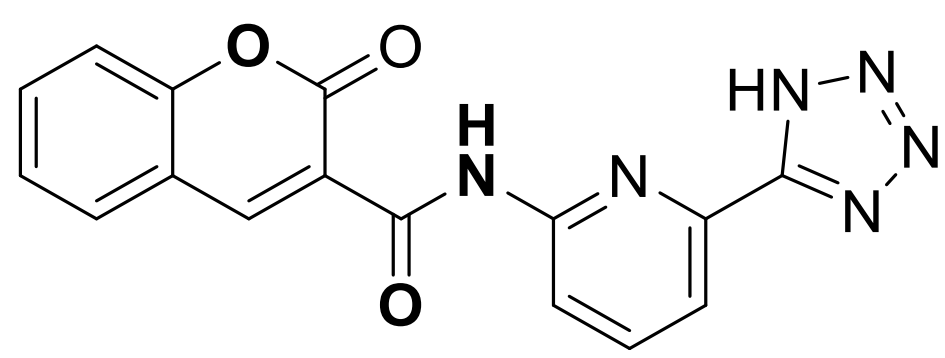	334.29	40% Inhibition against AD Seeding on average
**M11**	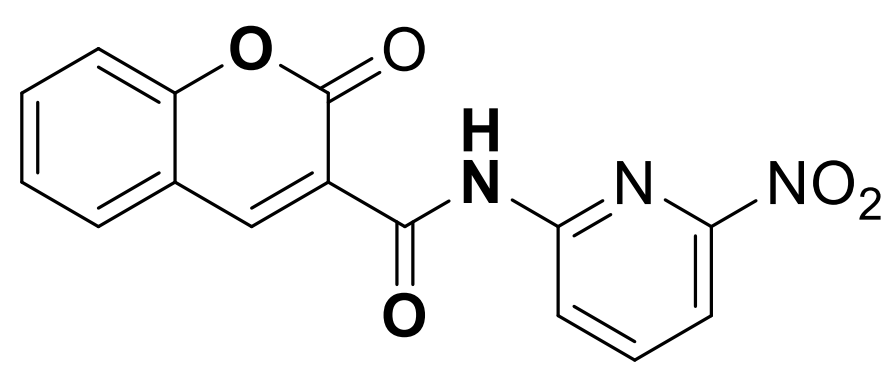	311.25	No notable effect on seeding
**M12**	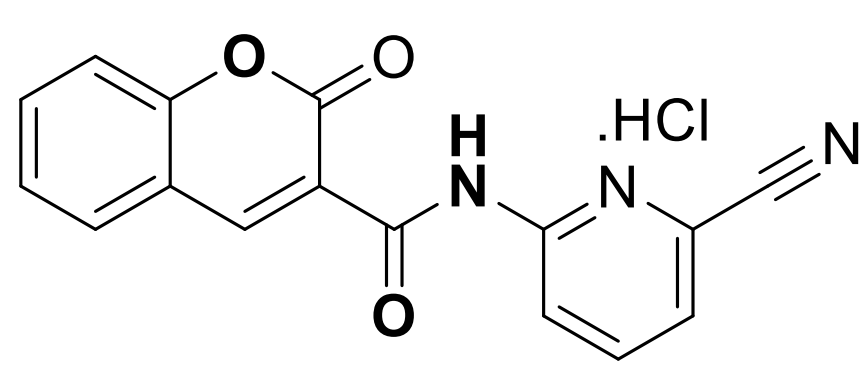	327.72	85% Inhibition against AD Seeding on average
**M13**	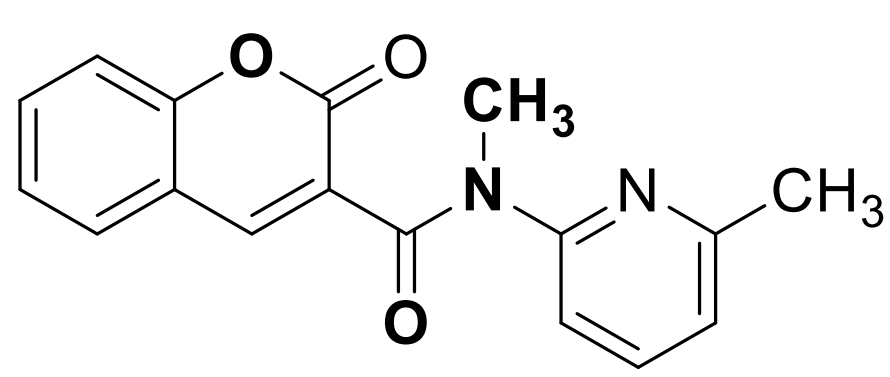	294.31	10% Increase in AD Seeding on average

Compound ID	Structure	Molecular Weight	Biological Data
**M14**	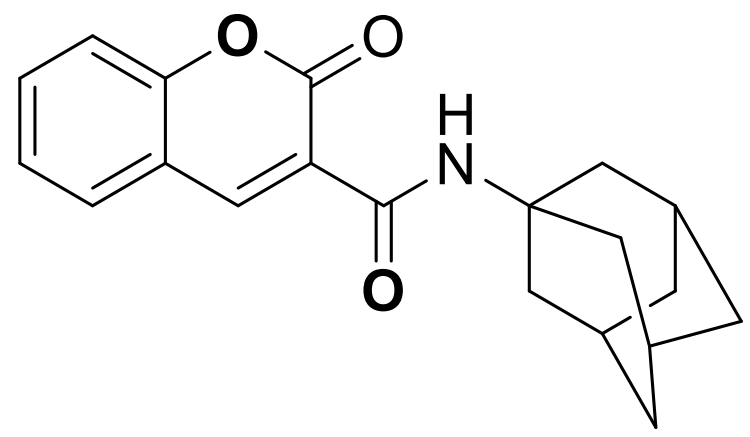	323.39	No notable effect on AD seeding
**M15**	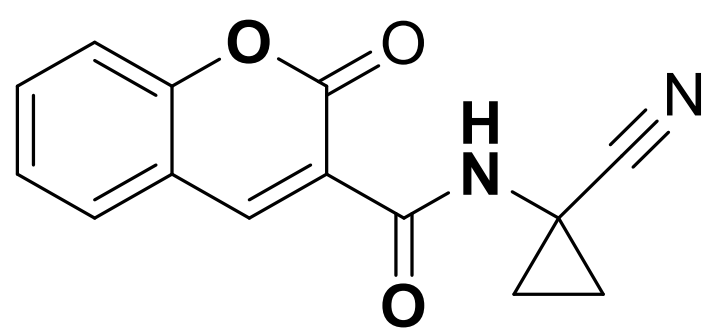	254.24	35% Inhibition against AD Seeding on average
**M16**	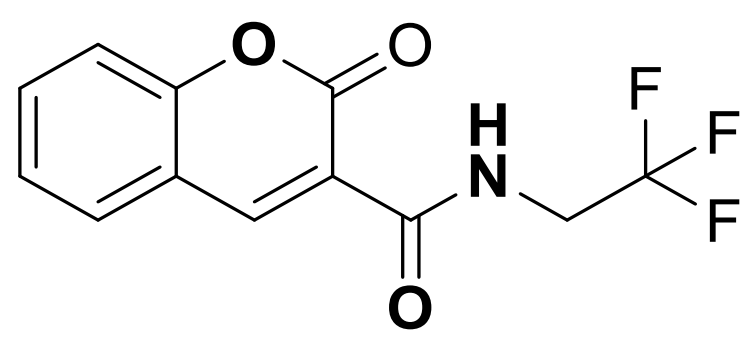	271.19	35% Inhibition against AD Seeding on average
**M17**	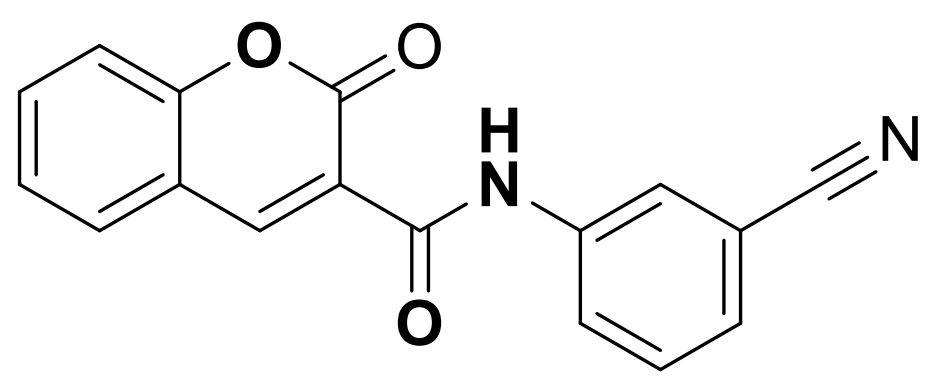	290.27	No notable effect on seeding
**M18**	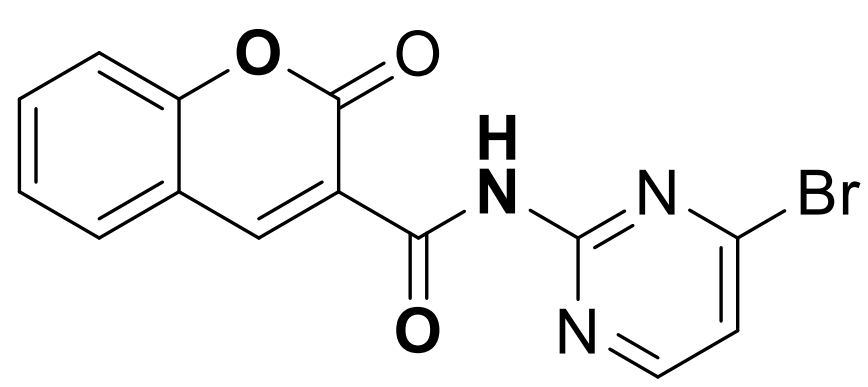	346.14	No notable effect on seeding
**M19**	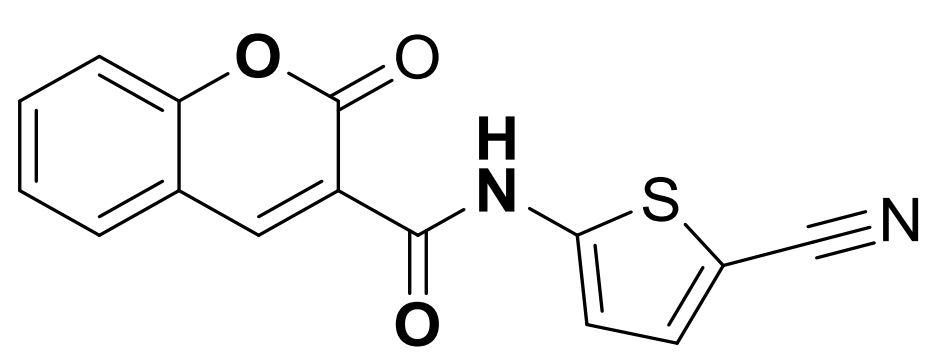	296.30	40% Inhibition against AD Seeding on average
**M20**	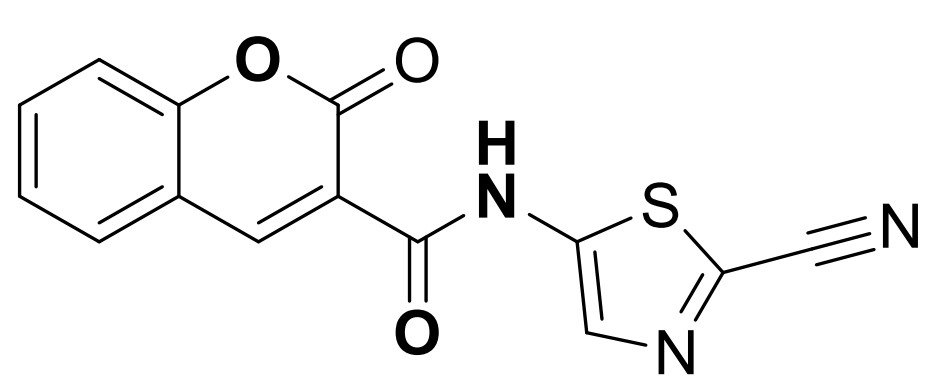	297.28	50% Inhibition against AD Seeding on average

Compound ID	Structure	Molecular Weight	Biological Data
**M21 'PT-46'**	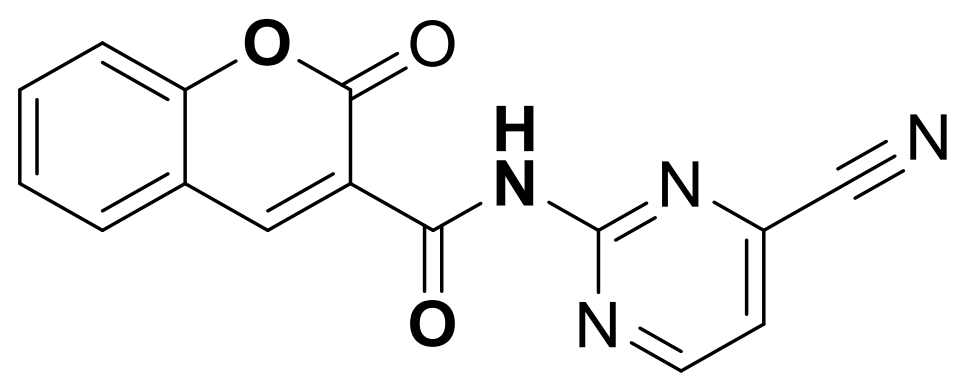	292.25	20% Inhibition against AD Seeding on average
**M22**	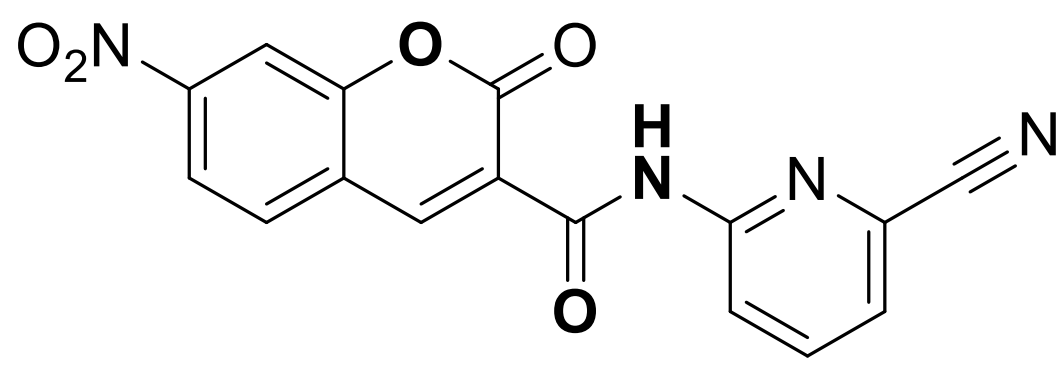	336.26	30% Increase in AD Seeding on average
**M23**	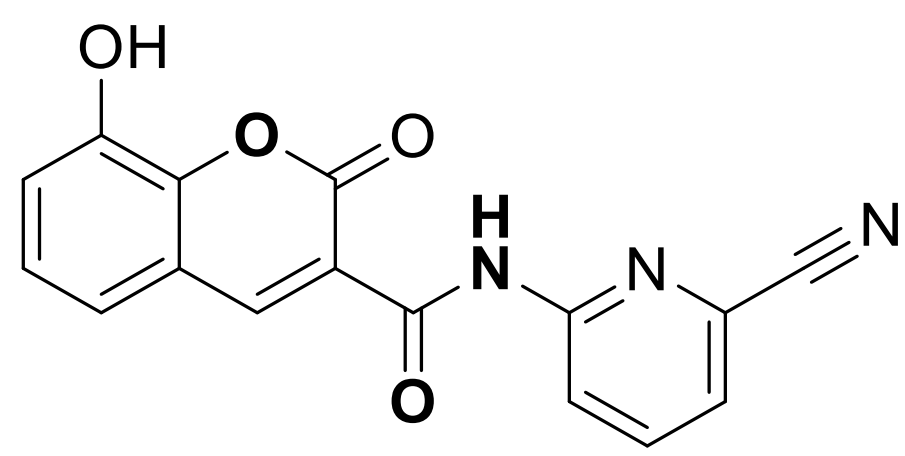	307.26	50% Inhibition against AD Seeding on average
**M24**	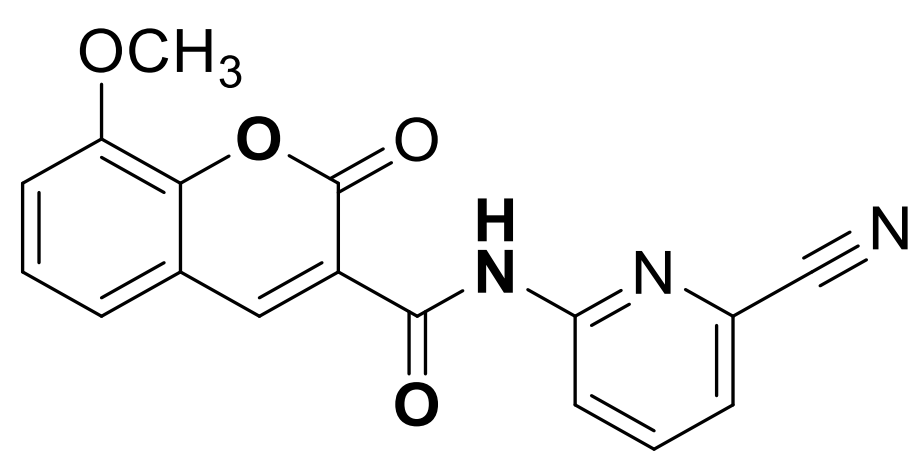	321.29	No notable effect on seeding
**M25**	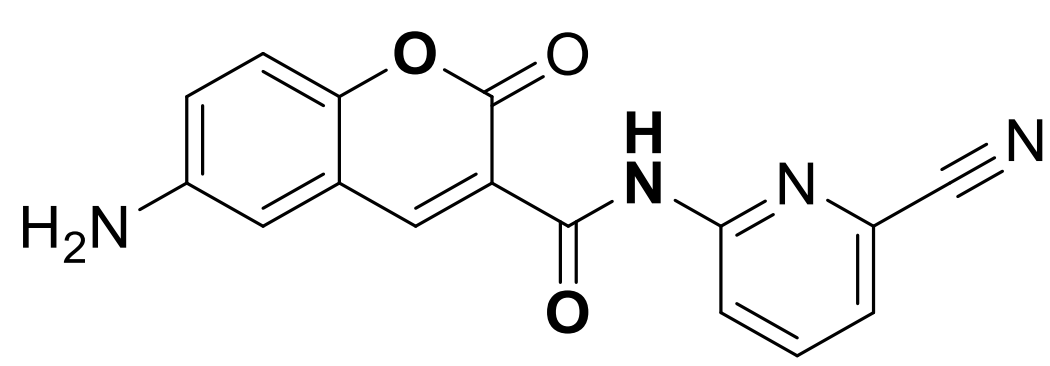	306.28	50% Inhibition against AD Seeding on average
**M26**	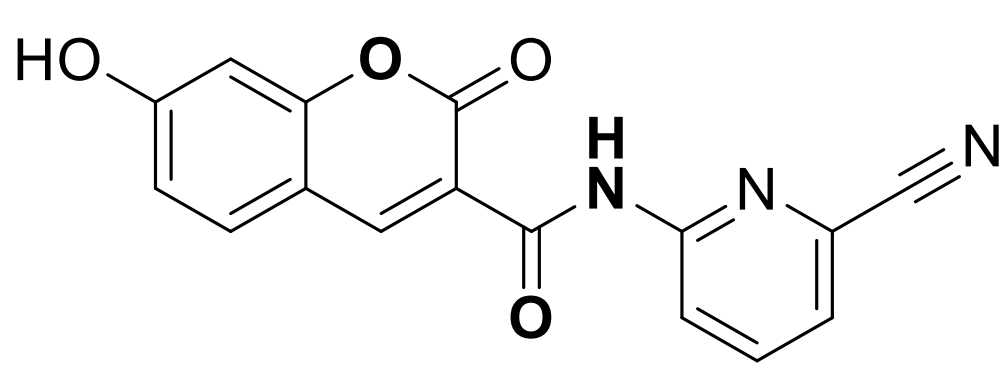	307.26	55% Inhibition against AD Seeding on average
**M27**	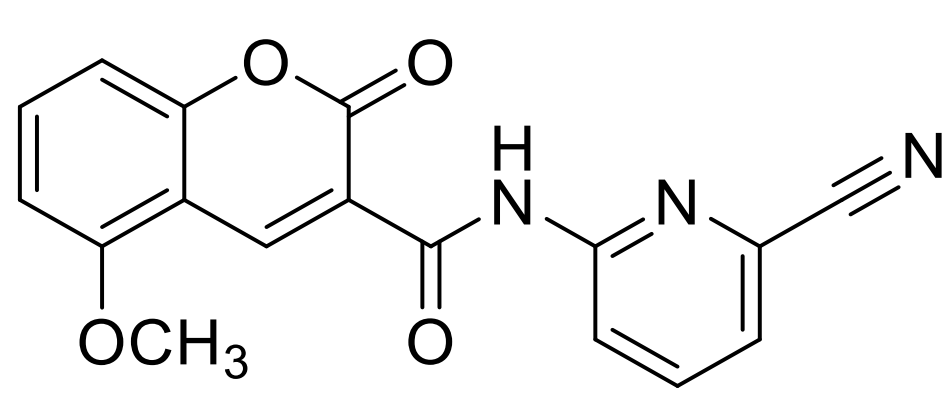	321.29	40% Inhibition against AD Seeding on average

Compound ID	Structure	Molecular Weight	Biological Data
**M28**	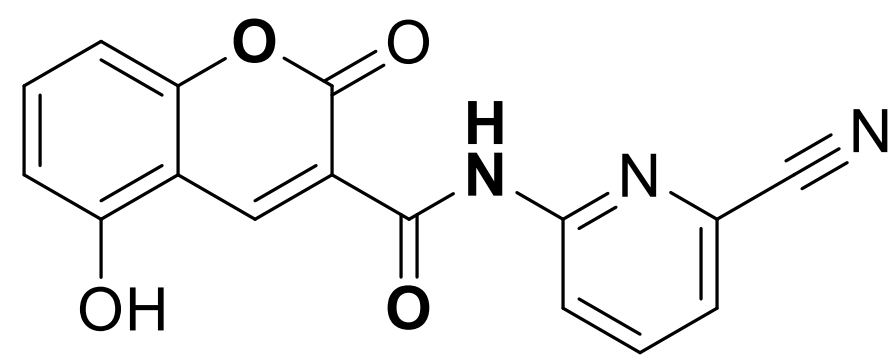	307.26	No notable effect on seeding
**M29**	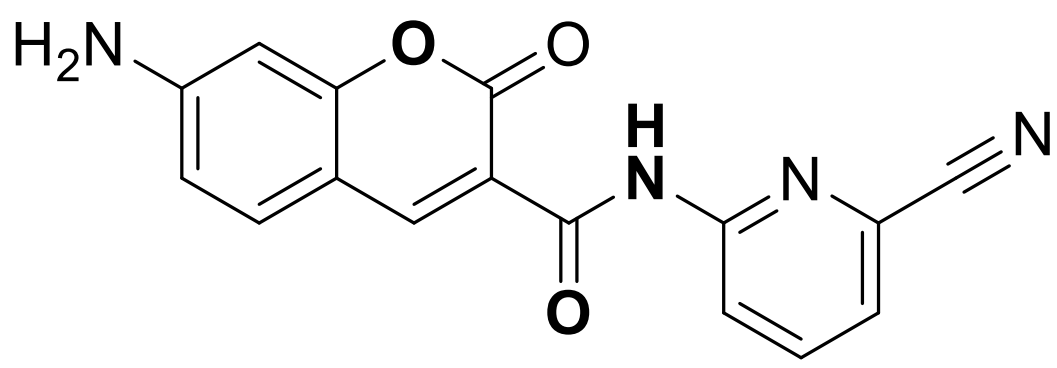	306.28	50% Increase in AD Seeding on Average
**M30**	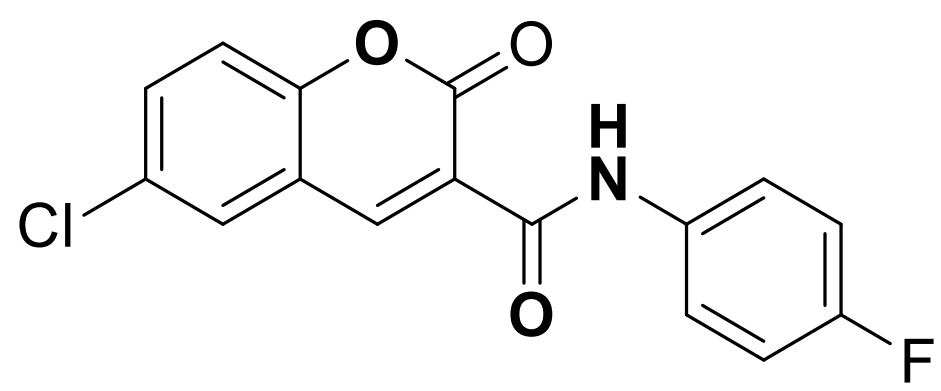	317.70	20% Increase in AD Seeding on average
**M31**	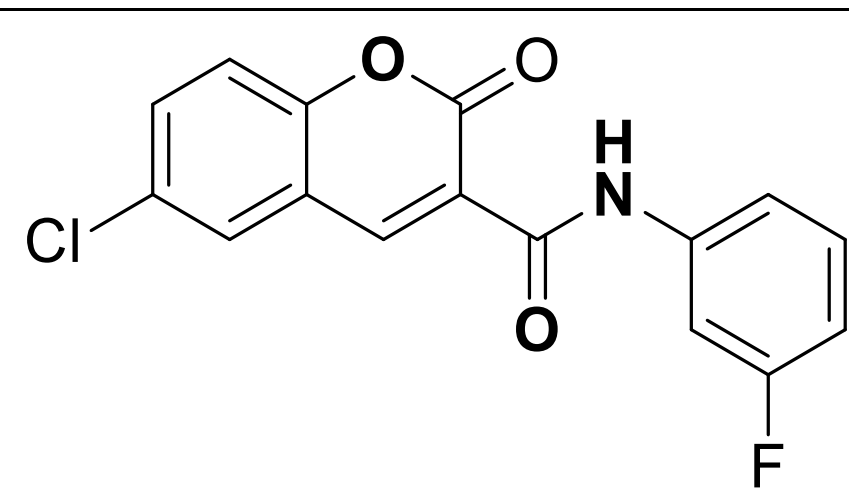	317.70	30% Inhibition against AD Seeding on average
**M32**	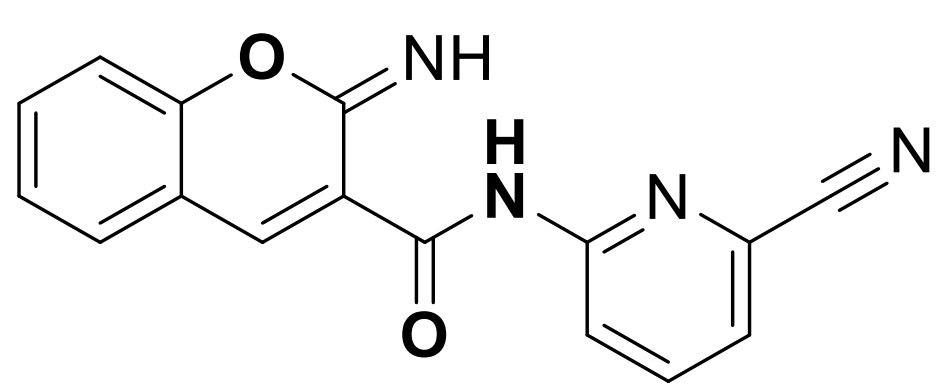	290.28	50% Inhibition against AD Seeding on average
**M33**	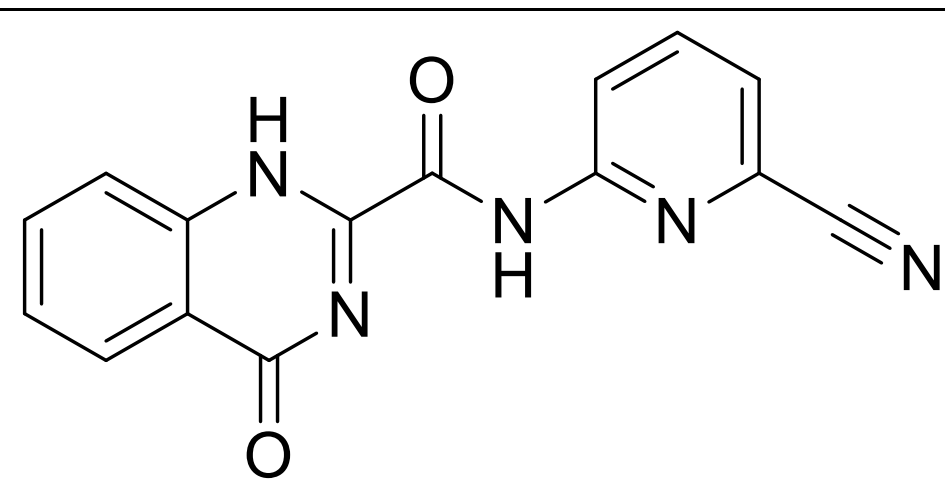	291.27	No notable effect on seeding
**M34**	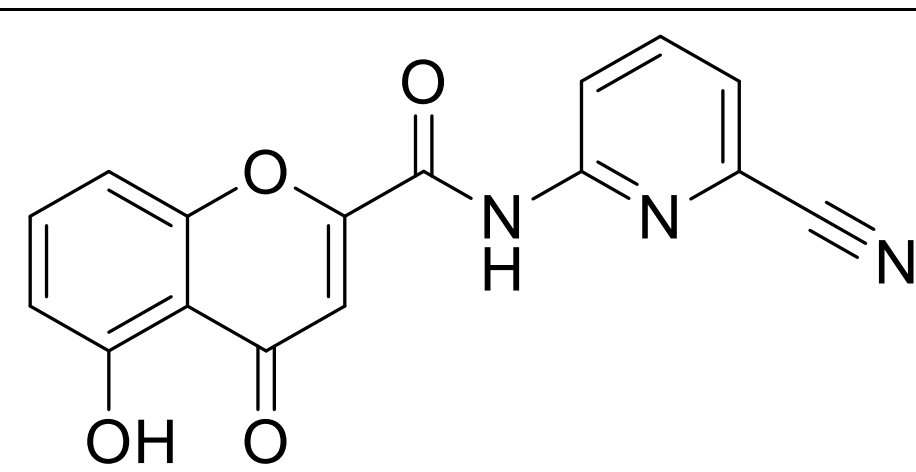	291.26	No notable effect on seeding

Compound ID	Structure	Molecular Weight	Biological Data
**M35**	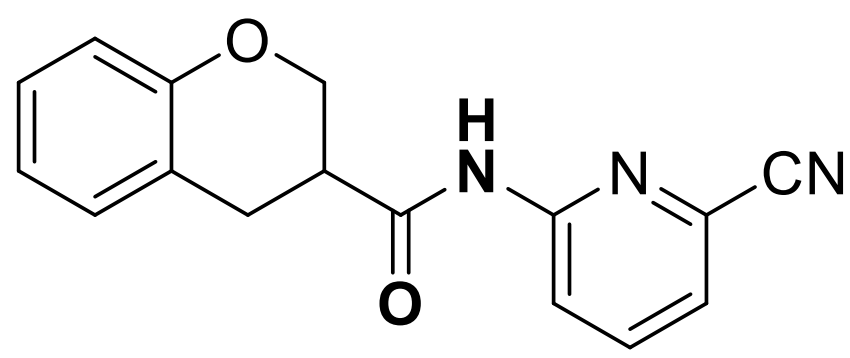	279.2990	No notable effect on seeding
**M36**	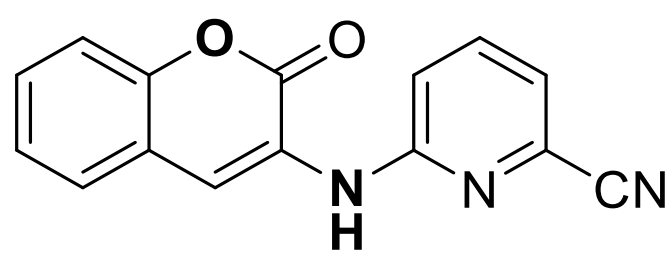	263.2560	No notable effect on seeding
**M37 'PT-243'**	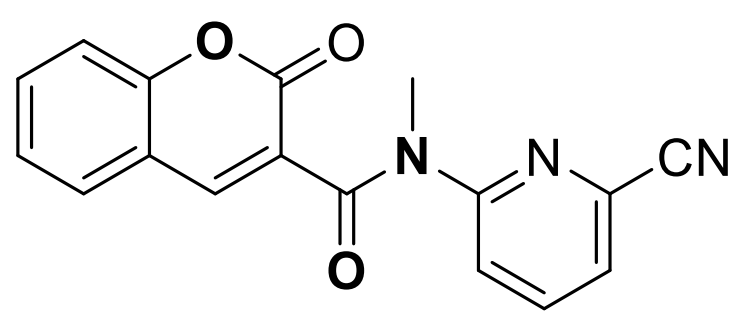	305.2930	No notable effect on seeding
**M38**	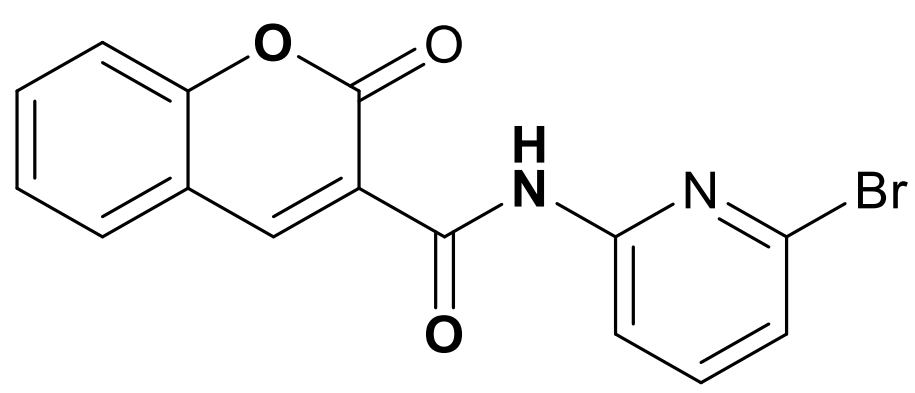	345.152	30% Increase in AD Seeding on average
**M39**	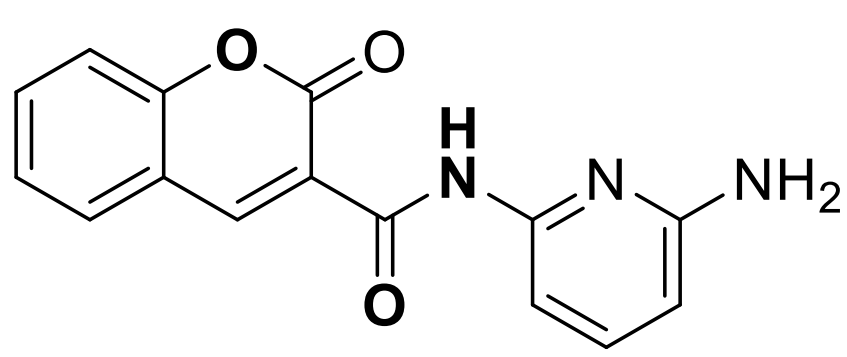	281.271	40% Inhibition against AD seeding on average
**M40**	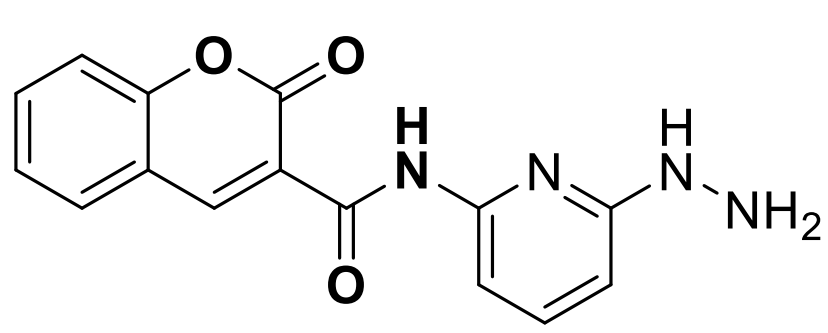	296.286	70% Inhibition against AD seeding on average
